# The Impact of Preoperative Nutritional Status on the Incidence of Anastomotic Leaks in Colorectal Surgery

**DOI:** 10.7759/cureus.104329

**Published:** 2026-02-26

**Authors:** Ulla Elfadil, Abdulrahman Al-Majmuei, Mohammad Alatoom, Sarah Juma, Abrar Shukralla

**Affiliations:** 1 Otolaryngology, Doncaster and Bassetlaw Teaching Hospitals NHS Foundation Trust, Doncaster, GBR; 2 Surgery, Royal College of Surgeons in Ireland, Al Muharraq, BHR; 3 Internal Medicine, Royal College of Surgeons in Ireland, Al Muharraq, BHR

**Keywords:** anastomosis leak, colorectal cancer, lower gi or colorectal surgery, nutritional status, nutritional support

## Abstract

Anastomotic leaks (ALs) are among the most serious complications following colorectal surgery, contributing to significant morbidity, reoperation, and prolonged hospitalisation. Poor preoperative nutritional status has been proposed as a modifiable risk factor; however, evidence from studies remains inconsistent. This systematic review, conducted according to Preferred Reporting Items for Systematic Reviews and Meta-Analyses (PRISMA) 2020 guidelines and registered in the International Prospective Register of Systematic Reviews (PROSPERO) (CRD420251034523), synthesised findings from 32 eligible studies, including randomised controlled trials, cohort studies, and case-control designs, published between 2005 and 2025. Across 474 initially identified articles, nutritional status was assessed using serum albumin, body mass index (BMI), Subjective Global Assessment (SGA), Nutritional Risk Index (NRI), or Prognostic Nutritional Index (PNI). AL incidence ranged from 2.8% to 11.3%, with hypoalbuminaemia and low nutritional indices consistently associated with increased risk. Several studies have suggested that nutritional optimisation, particularly immunonutrition and enteral support initiated 7-14 days preoperatively, improves secondary outcomes, such as wound infection rates, hospital stay, and overall morbidity. However, reductions in leak incidence are less consistent. The certainty of evidence linking poor nutritional status to leak risk was rated as moderate, while the evidence for nutritional interventions was rated as low due to heterogeneity and small sample sizes. Perioperative factors, including operative time, intraoperative blood loss, transfusion, and steroid use, were also significant contributors to leak risk. Overall, nutritional status is a key, modifiable predictor of AL; however, integration with surgical and perioperative optimisation is essential. High-quality multicentre trials are needed to define optimal nutritional strategies and establish standardised risk assessment tools for clinical practice.

## Introduction and background

Anastomotic leaks (ALs) are among the most serious and frequent complications following colorectal surgery, leading to increased morbidity and prolonged hospital stays. These leaks occur when the surgical connections between the two segments of the gastrointestinal tract fail to heal properly, resulting in leakage of contents into the abdominal cavity [1]. The incidence of AL varies depending on the type of surgery, but it remains a primary concern in colorectal surgeries, with significant implications for patient outcomes and healthcare systems.

In recent years, there has been growing interest in the role of nutritional status as a potential factor influencing the development of AL. Malnutrition, often characterised by low body mass index (BMI), hypoalbuminaemia, or poor preoperative nutrition scores, has been known to impair wound healing and immune function. These deficiencies may weaken the body’s response to healing surgical sites effectively, potentially increasing susceptibility to postoperative complications [2]. While several studies have explored the relationship between nutritional status and AL, findings have been inconsistent, highlighting the need for a comprehensive synthesis of the current evidence.

This systematic review aims to assess the association between nutritional status and the incidence of AL following colorectal surgery. Specifically, it seeks to determine whether poor nutritional indicators (e.g., low BMI, hypoalbuminaemia, or malnutrition screening scores) are associated with an increased risk of AL. By synthesising existing evidence, the review will also explore whether nutritional optimisation could be a viable strategy for reducing leak rates and improving postoperative outcomes.

## Review

Methods

**Figure 1:**
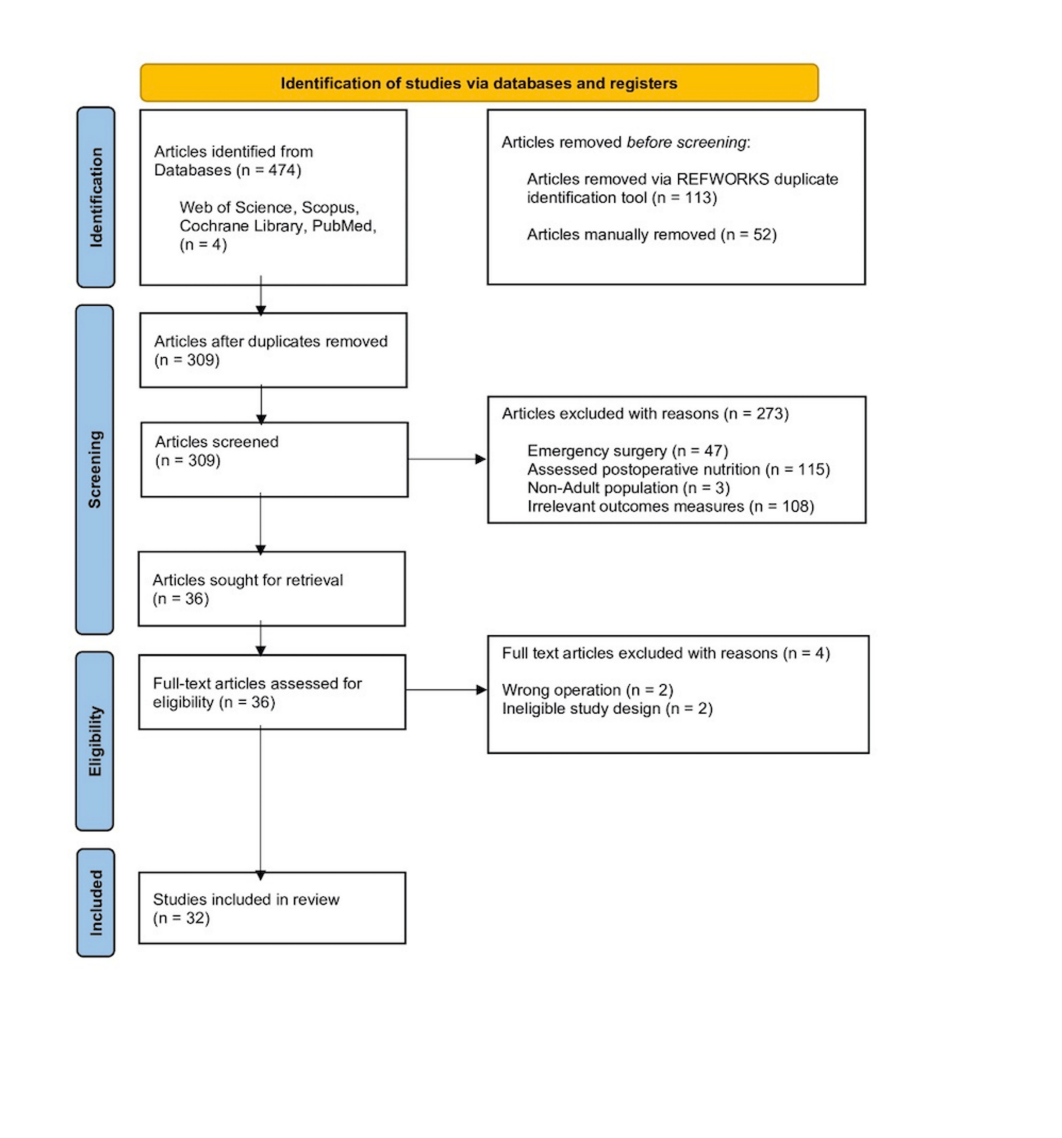
PRISMA flow diagram of the study selection process

This systematic review was conducted in accordance with the Preferred Reporting Items for Systematic Reviews and Meta-Analyses (PRISMA) 2020 guidelines (Figure 1). The protocol was registered with the International Prospective Register of Systematic Reviews (PROSPERO), under the registration ID CRD420251034523. The protocol was finalised before screening to ensure transparency, reproducibility, and adherence to best practices.

Studies were eligible for inclusion if they involved adult patients aged 19 years or older undergoing elective colorectal surgery with a primary bowel anastomosis. Eligible studies assessed preoperative nutritional status, either at baseline or as part of a targeted intervention. Malnutrition was defined according to one or more standardised criteria, including BMI < 18.5, serum albumin < 3.5 g/dL, Subjective Global Assessment (SGA) Grade B or C, NRI < 97.5, or Nutritional Risk Screening 2002 (NRS 2002) score of ≥3.

The primary outcome of interest was the incidence of AL, as defined by each study. Secondary outcomes included postoperative mortality, length of hospital stay, incidence of surgical site infection (SSI), need for reoperation or intervention, and time to diagnosis of AL. Eligible study designs included randomised controlled trials, cohort studies, and case-control studies. Only articles published in English between January 2005 and April 2025 were considered. Exclusion criteria included emergency procedures, non-colorectal anastomoses, studies involving paediatric populations, and publications such as case reports, editorials, narrative reviews, animal studies, conference abstracts, or non-English papers.

We systematically searched four electronic databases, including PubMed, Web of Science, Scopus, and the Cochrane Library. Searches were conducted using a combination of Medical Subject Headings (MeSH) and free-text terms related to ALs, nutritional status, and colorectal surgery. Key search terms included “colorectal surgery”, “colon surgery”, “rectal surgery”, “bowel resection”, “anastomotic leak”, “anastomotic dehiscence”, “malnutrition”, “nutritional assessment”, “preoperative nutritional status”, “oral nutritional supplements”, “enteral nutrition”, “parenteral nutrition”, “immunonutrition”, “serum albumin”, and “body mass index”. Boolean operators were used to combine search terms, refining the sensitivity and specificity of the search. This core strategy was applied across all four aforementioned databases, with minor modifications to accommodate the indexing systems and syntax requirements unique to each platform. We also manually screened the reference lists of all full-text included articles to identify additional relevant studies.

Search results were imported into the Rayyan (Rayyan Systems Inc., Cambridge, MA, USA) web-based screening platform. Title and abstract screenings were conducted independently by two reviewers. Articles that met the inclusion criteria or were unclear were assessed at the full-text level. Discrepancies were resolved through discussion or, if needed, adjudicated by a third reviewer.

A standardised extraction form was developed and piloted for consistency. For each study, the following data were collected: title, first author, publication year, country, study design, sample size, age (mean, median, including range if available), sex distribution, inclusion and exclusion criteria, and participant comorbidities. Details on surgical approach (laparoscopic or open), type of anastomosis, and preoperative nutritional assessment methods were recorded. Where applicable, information on nutritional interventions, including type (oral, enteral, parenteral, or immunonutrition) and duration, was also extracted.

For the primary outcome, the incidence of AL was documented, including any association with nutritional status or intervention. The methods used to confirm AL (clinical, radiological, or intraoperative) were also recorded. Secondary outcomes included postoperative mortality related to leaks, hospital stay duration, need for reoperation, time to diagnosis, and SSIs. Where available, effect sizes (odds ratios, risk ratios, or hazard ratios) with confidence intervals and p-values were extracted.

The methodological quality of all included studies was assessed independently by two reviewers. For cohort and case-control studies, the Newcastle Ottawa Scale (NOS) was used. This tool evaluates three domains, including the selection of study groups, comparability of these groups, and the ascertainment of exposure or outcome. A maximum of nine points could be awarded, with higher scores indicating better methodological quality. For any included randomised controlled trials, the Cochrane Risk of Bias tool (RoB 2) was used, which assesses bias across five domains, including the randomisation process, deviations from intended interventions, missing outcome data, measurement of the outcome, and selection of the reported result.

Disagreements between reviewers during quality assessments were resolved through discussion. Where consensus could not be reached, a third reviewer was consulted. The results of these assessments were used to inform the interpretation of study findings but did not determine eligibility for inclusion.

Potential reporting bias was assessed by comparing reported outcomes with those in trial registries, published protocols, or methods sections where available. The certainty of evidence for the primary and key secondary outcomes was assessed using the grading of Recommendations, Assessment, Development and Evaluation (GRADE) framework. This considers study limitations, inconsistency, indirectness, imprecision, and potential publication bias. Certainty of evidence was rated as high, moderate, low, or very low. 

Results

A total of 474 articles were retrieved through database searching. After removing 165 duplicates (113 via RefWorks (Clarivate, Philadelphia, PA, USA) and 52 via manual review in Rayyan), 309 unique records were identified for screening. Of these, 273 were excluded based on title and abstract review: 47 assessed emergency surgery, 115 focused on postoperative nutrition, 3 included non-adult populations, and 108 did not report relevant outcomes. Thirty-six full-text articles were left to be assessed, of which 4 were excluded due to the use of an ineligible surgical procedure and improper study design, resulting in 32 studies included in the final review. 

Of the 32 included studies, 15 were retrospective cohort studies, 7 were prospective cohort studies, 6 were randomised controlled trials, and the remainder consisted of case-control designs and multicentre observational surveys. Studies were conducted across Europe, Asia, North America, and Oceania, and all were published between 2005 and 2025, in accordance with our protocol. Sample sizes ranged from under 100 to over 1,000 participants. Patient ages were generally in the range of 40-75 years, with relatively balanced sex distributions. Commonly reported comorbidities included diabetes, cardiovascular disease, obesity, and malignancy. 

All studies assessed preoperative nutritional status using at least one standardised method. The most commonly used indicators were serum albumin (in 28 studies), BMI (in 25 studies), SGA (in 7 studies), NRI (in 6 studies), and NRS 2002 (in 4 studies). Fifteen studies (46.9%) reported a preoperative nutritional intervention (oral nutritional supplements, immunonutrition, enteral feeding, carbohydrate loading, or total parenteral nutrition (TPN)). The remaining 17 studies (53.1%) evaluated nutritional status preoperatively without a reported nutritional intervention. Duration of intervention, when reported, ranged from 7 to 28 days. The remaining seven studies measured and monitored nutritional levels preoperatively without intervening. 

All 32 studies reported the incidence of AL, which ranged from 2.8% to 11.3%. Leak rates were generally higher in patients identified as malnourished. For instance, Ionescu et al. found that patients with moderate hypoalbuminaemia had an odds ratio of 6.65 for developing a leak (95% CI: 2.37-18.62) [3]. Detection of AL was most commonly based on clinical signs (fever, abdominal pain, drainage), laboratory markers (elevated C-reactive protein, leucocytosis), and radiological confirmation, typically with a contrast-enhanced computed tomography. A complete summary of nutritional markers and leak outcomes is provided in Table 1. 

**Table 1:** Associations between preoperative nutritional markers and anastomotic leak risk

Author (year)	Nutritional marker(s) assessed	Leak incidence (%) (high vs low nutritional status)	Effect size (odds ratio, risk ratio, etc.)	p-value	Reference
Ionescu et al. (2013)	Serum albumin (hypoalbuminaemia <3.5 g/dL; severe <2.5 g/dL)	Overall anastomotic leak (AL) rate: 5.55%; normal albumin: 2.3%; hypoalbuminaemia: 13.3%; severe hypoalbuminaemia: 42.9%	Moderate hypoalbuminaemia: OR 6.65 (95% CI: 2.01-21.96); severe hypoalbuminaemia: OR 24.75 (95% CI: 6.75-90.67)	Moderate: 0.001; severe: <0.001	[3]
Telem et al. (2010)	Preoperative serum albumin <3.5 g/dL	Overall AL: 2.6% (90/3501)	OR 2.8 (95% CI 1.3-5.1)	p = 0.03	[4]
Golda et al. (2020)	Preoperative serum albumin	Overall AL rate: 9.4% (44/470)¹	OR 0.927 (95% CI: 0.88-0.97)	0.004	[5]
Suding et al. (2008)	Preoperative serum albumin (≥3.5 g/dL)	Low albumin (<3.5 g/dL): 10/143 patients (7%); normal/higher albumin (≥3.5 g/dL): 13/519 (2.5%); overall AL: 3.6% (24/672)	OR 2.56 (95% CI 1.07-6.16)	p = 0.04	[6]
Suzuki et al. (2020)	Preoperative serum albumin (≥4 g/dL)	Overall AL: 15.4% (21/136)	OR 0.29 (95% CI 0.07-1.16)	p = 0.081	[7]
Shimura et al. (2018)	Preoperative serum albumin	Overall AL rate: 5.6% (11/196)	Preoperative serum albumin <3.2 g/dL: OR 7.53 (95% CI 1.60-55.80)	p = 0.0095	[8]
Fransson et al. (2016)	Preoperative serum total protein concentration; preoperative serum albumin concentration	Overall AL rate: 8.4%; lower preoperative serum total protein associated with increased AL risk¹	All AL: pre-op serum total protein OR 0.86 per g/dL (95% CI: 0.4-0.8); major AL: pre-op serum total protein OR 0.86 per g/dL (95% CI: 0.7-0.98); stapled anastomosis OR 2.1 (95% CI: 1.1-4.2)	All AL: <0.0001; major AL: protein 0.004, stapled 0.03	[9]
Zhang et al. (2025)	Onodera Prognostic Nutritional Index (OPNI), neutrophil-to-lymphocyte ratio (NLR), platelet-to-lymphocyte ratio (PLR)	Overall AL rate: 11.28% (49/434); lower OPNI (<46.6), higher NLR (≥2.98), and higher PLR (≥158.15) associated with increased AL risk¹	OPNI: OR 0.705 (95% CI: 0.641-0.775); NLR: OR 1.628 (95% CI: 1.221-2.172); PLR: OR 0.994 (95% CI: 0.989-0.999)	OPNI: 0.012; NLR: 0.024; PLR: 0.031	[10]
Kwag et al. (2014)	Nutritional Risk Screening 2002 (NRS 2002) score	High risk (NRS ≥ 3 ): 91% (9/99); low risk (NRS ≤ 3 ): 3.1% (8/253)	OR 3.06 (CI 1.148-8.185)	p = 0.027	[11]
Wei et al. (2024)	Prognostic Nutritional Index (PNI)	Overall AL rate: 19.35% (24/124); lower PNI is associated with increased AL risk¹	Area under the curve (AUC) (PNI): 0.625 (95% CI: 0.52-0.73)	0.007	[12]
Brisinda et al. (2022)	PNI	Low PNI: 18.2% (PNI < 40), 29/130 patients vs adequate PNI: 6.6% (PNI ≥ 40), 32/453 patients	Not reported	p = 0.0001	[13]
Ostermann et al. (2019)	Enhanced recovery program (multimodal pathway including early oral feeding; no specific nutritional biomarker assessed)	0% (0/75) vs 6.7% (5/75)	Not reported	0.01	[14]
Paliogiannis et al. (2021)	C-reactive protein-to-albumin ratio (CAR)	Overall AL rate: 7.5%; higher CAR (>46) linked to increased AL risk¹	OR 1.045 (95% CI: 1.031-1.058)	<0.0001	[15]
Do Woong Choi et al. (2023)	Preoperative serum albumin and total protein levels measured 1 week before surgery. Preoperative oral immunonutrition (Oral Impact: arginine, omega-3 fatty acids, nucleotides)	Overall AL: 4.4% (16/361)	Not reported	Albumin: p = 0.4457; protein: p = 0.6245	[16]
Reisinger et al. (2015)	Short Nutritional Assessment Questionnaire (SNAQ), score ≥ 3 is defined as preoperative malnutrition	Overall leak: 14.9%	High SNAQ score(≥3): OR = 2.33 (95% CI 0.90-6.00)	p = 0.08	[17]
Manzanares Campillo et al. (2017)	Preoperative oral immunonutrition (Oral Impact: arginine, omega-3 fatty acids, nucleotides)	AL: YES-IN 11.9% (5/42) vs NO-IN 16.7% (7/42). Overall infectious complications: YES-IN 33.3% vs NO-IN 40.5%	Not reported	AL: NS (p = 0.533); overall infections: NS (p = 0.489)	[18]
Thornblade et al. (2017)	Preoperative oral immunonutrition (Impact)	Adverse events (including AL): 7.1%	Serious adverse events (SAEs): RR 0.76 (95% CI: 0.49-1.16); prolonged length of stay (PLOS): RR 0.77 (95% CI: 0.58-1.01)	SAE: p = 0.19; PLOS: p = 0.05	[19]
Moya et al. (2016)	Perioperative immunonutrition (arginine, omega-3 fatty acids, nucleotides) in normo-nourished patients	AL not reported; no difference in overall postoperative morbidity or mortality between immunonutrition and control groups	Not reported	AL not reported; wound infection: p = 0.006	[20]
Braga et al. (2002)	Immunonutrition intervention: preoperative oral arginine- and n-3 fatty-acid-enriched formula (5 days pre-op)	AL not separately reported	No effect size reported for AL	Not reported for AL	[21]
Horie et al. (2006)	Preoperative enteral immunonutrition (arginine, nucleotides, omega-3 fatty acids) administered for 5 days pre-op	AL not reported	Relative reduction in SSI. Absolute risk reduction: 11.8%	<0.05	[22]
Finco C (2007)	Perioperative enteral immunonutrition enriched with arginine, omega-3 fatty acids, and RNA for 6 days pre-op and resumed post-op day 2 vs low-fibre diet	Not reported	Not reported	Not reported	[23]
Oguz et al. (2007)	SGA system used to classify patients' nutritional status (normal, moderate malnutrition, severe malnutrition)	Overall AL: 0% major leaks, 1% minor leaks in the glutamine group. In the control group, 0% major leaks and 4% minor leaks	Not reported	Not reported	[24]
Peters et al. (2018)	Perioperative lipid-enriched enteral nutrition (continuous tube feeding from 3-h pre-op to 6-h post-op) vs no perioperative nutrition	AL: 9% (12/132) intervention vs 8% (11/133) control	RR 1.01 (95% CI 0.94-1.09)	0.81	[25]
Younan et al. (2025)	Preoperative oral immunonutrition (Oral Impact). Albumin and pre-albumin were measured	Received immunonutrition (4.8%) vs did not receive immunonutrition (5.2%)	OR 0.58 (95% CI 0.22-1.56)	p = 0.58	[26]
Guo et al. (2016)	Preoperative serum albumin, C-reactive protein (CRP). Preoperative nutritional therapy (NT) with enteral nutrition was given	Received preoperative therapy, 2.3% (1/44): high nutritional status, 17.9% (7/39). Did not receive preoperative therapy: low nutritional status	Not reported	p = 0.023	[27]
Salinas et al. (2012)	Preoperative total parenteral nutrition (TPN) vs no TPN	AL: 5.4% (TPN) vs 2.8% (no TPN)	Not reported for leak	0.356	[28]
Xu and Kong (2020)	Patient-Generated Subjective Global Assessment (PG-SGA) score; postoperative albumin	Overall AL rate: 14.65% (56/382). Lower postoperative albumin and higher PG-SGA score associated with increased AL risk¹	Post-op albumin: OR 1.137 (95% CI: 1.004-1.287); PG-SGA: OR 22.988 (95% CI: 6.541-80.792)	Albumin: 0.044; PG-SGA: <0.001	[29]
Yamamoto et al. (2020)	Serum albumin and CRP measured before and after preoperative eternal nutrition	Enteral nutrition group: 4% (1/24); control: 12.5% (3/24)	Not reported	Overall sepsis complication (including leaks) p = 0.04	[30]
Asteria et al. (2008)	Malnutrition defined by weight loss >10% within six months and low BMI	Malnourished (30%) vs non-malnourished (13%)	OR 2.8 (95% CI 1.52-5.18)	p = 0.001	[31]
Sørensen et al. (1999)	Lifestyle factors (smoking status; alcohol abuse), not biochemical nutritional markers	Overall AL rate: 15.9% (53/333). Higher leak incidence observed in smokers and alcohol abusers compared with non-smokers and abstainers	Smoking: RR 3.18 (95% CI 1.44-7.00); alcohol abuse: RR 7.18 (95% CI 1.20-43.01)	Smoking: p < 0.05; alcohol abuse: p < 0.05	[32]
Drautz et al. (2015)	Intraoperative fluid administration (low vs high volume; median cutoff 0.15 mL/kg/min)	AL: 3% (low-fluid group) vs 6% (high-fluid group)	Not reported	0.26	[33]
Todurov et al. (2023)	Plasma albumin level	Enhanced recovery after surgery (ERAS) group: 1 out of 56 patients (1.8%); control: 3 out of 50 patients (6%)	Not reported	Not reported	[34]

Some studies investigated the impact of targeted nutritional support. In a retrospective cohort study by Salinas et al., patients who received preoperative TPN had a leak rate of 5.4%, compared to 2.8% in those who did not receive TPN (p = 0.18) [28]. While the difference was not statistically significant, the study noted improved postoperative outcomes in malnourished patients with nutritional optimisation [5]. Other studies evaluating immunonutrition or oral supplements reported similar trends, though effect sizes and p-values varied. These findings supported the potential benefit of nutritional optimisation but underscore the need for more robust controlled trials. 

Based on quality assessments using the NOS for cohort studies and the Cochrane RoB 2 for randomised controlled trials, 21 studies were rated as high quality, 11 as moderate quality, and 3 as low quality. High-quality studies consistently reported clear inclusion criteria, valid nutritional assessments, and adjustment for confounders. Moderate- and low-quality studies often had retrospective designs and lacked clarity in outcomes assessment or confounder control. No studies were excluded solely based on methodological quality. 

We have identified no registered protocols for the included observational studies, which limits our ability to rule out selective outcomes reporting. Among the six randomised controlled trials, four had accessible trial registries and reported their prespecified primary outcomes. Based on the GRADE assessment, the certainty of evidence supporting an association between poor nutritional status (particularly hypoalbuminaemia and low prognostic nutritional indices) and increased risk of AL was rated as moderate. Certainty was downgraded due to reliance on predominantly observational studies. Evidence for the effectiveness of preoperative nutritional interventions was rated as low certainty, reflecting inconsistency across trials and small sample sizes. 

Secondary outcomes were reported across nearly all included studies and revealed a consistent pattern of worse postoperative results in patients who developed AL. Thirty-two studies reported on mortality outcomes, with 30-day mortality in leak patients ranging from 1.8% to 7.2%, often significantly higher than in those without leaks. Leak-related complications also prolonged hospitalisation. For instance, Paliogiannis et al. found that patients with leaks had a mean hospital stay of 21.3 ± 11.4 days, compared to 11.6 ± 5.7 days in patients without leaks [15]. Reoperation or radiological intervention was commonly required, with rates ranging from 3.9% to over 10% [6]. A study reported a 6% reoperation rate among 470 patients, primarily in those with Grade C leaks [7]. The time to diagnosis of AL was reported in 32 studies, with most leaks detected within the first 7-10 days postoperatively. Golda et al. found the mean time to diagnosis was 8.5 days, with more severe cases presenting earlier [5]. Additionally, 31 studies documented SSIs, which were more frequent in patients who experienced leaks. For example, Ionescu et al. reported an overall infection rate of 2.38%, with a clear association between infection risk and poor nutritional status [3]. A complete summary of secondary outcomes is provided in Table 2. 

**Table 2:** Effects of preoperative nutritional interventions on anastomotic leak and surgical site infection

Author (year)	Type of intervention and duration	Leak incidence (intervention vs control)	Effect size	p-value	Reference
Salinas et al. (2012)	Preoperative total parenteral nutrition (TPN) for ≥7 days (range 7-28 days; median 9 days)	3.6% (3/56) vs 2.8% (5/179)	OR 2.52 (including line infections); OR 1.24 (excluding line infections)	p = 0.356 (leak incidence); p = 0.04 (total complications including line infections)	[28]
Braga et al. (2002)	Preoperative oral immunonutrition with arginine (12.5 g/L) and n-3 fatty acids (3.3 g/L) for 5 days before surgery; compared with standard isonitrogenous, isocaloric formula, or no supplementation	6% (3/50) vs 11% (6/50 control) and 10% (5/50 conventional)	Not reported for leak rate	Nutritional status (NS) for leak rate; p < 0.04 for infection rate reduction	[21]
Horie et al. (2006)	Preoperative enteral immunonutrition (arginine, nucleotides, omega-3 fatty acids), 5 days pre-op, 750 mL/day	Not reported	Not reported for leak rate	NS for leak rate; p < 0.05 for surgical site infection (SSI) reduction	[22]
Manzanares Campillo et al.(2017)	Preoperative oral immunonutrition (Oral Impact), 3 sachets/day for 8 days pre-op	11.9% (5/42) vs 16.7% (7/42)	Not reported	p = 0.533 (leak rate)	[18]
Moya et al. (2016)	Perioperative immunonutrition (arginine, omega-3, RNA) for 7 days preoperatively + 5 days postoperatively vs dietary advice only	4.9% (3/61) vs 3.3% (2/61)	RR 0.655 (95% CI 0.106-4.067)	p = 0.648	[20]
Ostermann et al. (2019)	Enhanced recovery program (ERP) with multimodal perioperative care: preoperative counselling, nutritional assessment and support (oral supplements twice daily for 7 days if NRS ≥3), oral carbohydrate loading, multimodal analgesia with systemic lidocaine, early feeding, and mobilisation	0% (0/75) vs 6.7% (5/75)	OR for reduced morbidity = 0.23 (95% CI 0.09-0.57)	p = 0.01 (anastomotic leak (AL))	[14]
Todurov et al. (2023)	2018 version of enhanced recovery after surgery (ERAS) at pre-, post-, and intraoperative stages. Preoperative high‑protein oral supplementation of 900-1200 kcal, 54-72 g protein, and albumin 20% for hypoalbuminaemia. Iron for anaemia	1.8% (1/56) vs 6% (3/50)	Not reported for leak rate	NS for leak rate; p < 0.001 for VAS score, opioid requirement, return of bowel function, post-op complications, length of hospital stay, resumption of fluid intake, and enteral feeding	[34]
Suzuki et al. (2021)	No nutritional intervention given, patient risk factor assessed and serum albumin	9.6 % (5/52) vs 19.0 % (16/84)	OR for leak with stoma = 0.05 (95 % CI 0.01-0.26)	p < 0.001 (protective effect of stoma)	[7]
Zhang et al. (2025)	No nutritional intervention; preoperative assessment of nutritional and inflammatory biomarkers (Onodera Prognostic Nutritional Index, OPNI; neutrophil-to-lymphocyte ratio, NLR; platelet-to-lymphocyte ratio, PLR) prior to rectal cancer surgery	Overall AL incidence: 11.28% (49/434)	Low OPNI, high NLR, and high PLR were independent predictors of AL on multivariate logistic regression	All predictors are statistically significant (p < 0.05). Combined model demonstrated good discrimination on ROC analysis	[10]
Paliogiannis et al. (2021)	No nutritional intervention; postoperative inflammatory biomarker assessment: C-reactive protein-to-albumin ratio (CAR) measured on postoperative day 4	Not reported as incidence groups; CAR compared between patients with vs without AL	AUC 0.825 (95% CI 0.786-0.859) for predicting AL; superior to CRP or albumin alone	p < 0.001 (ROC analysis)	[15]
Ionescu et al. (2013)	No nutritional intervention; preoperative nutritional status assessment: serum albumin measured preoperatively; hypoalbuminaemia defined as albumin <3.5 g/dL	Fistula incidence: 13.3% in hypoalbuminaemic patients vs 2.3% in patients with normal albumin	Moderate hypoalbuminaemia: OR 6.65 (95% CI 2.01-21.96); severe hypoalbuminaemia: OR 24.75 (95% CI 6.75-90.67)	p = 0.001	[3]
Golda et al. (2020)	No nutritional intervention; preoperative and perioperative risk factor assessment in patients undergoing elective ileocolic anastomosis	Overall AL rate: 9.4% (6.0% severe, 3.4% mild); leak incidence compared across risk-factor groups (not intervention vs control)	Preoperative low serum albumin identified as independent risk factor (effect size not numerically specified in abstract); suture oversewing protective	Albumin p = 0.004; smoking p = 0.005; blood transfusion p = 0.038; oversewing p < 0.001	[5]
Drautz et al. (2015)	Restrictive vs liberal intraoperative fluid administration according to ERAS protocol; patients divided by median intraoperative fluid rate (0.15 mL/kg/min)	3% (lower fluid group) vs 6%(higher fluid group)	Not reported	p = 0.26 (AL); p = 0.23 (overall complications)	[33]
Xu and Kong (2020)	No nutritional intervention; assessment of perioperative nutritional status (NRS 2002, PG-SGA, albumin) in rectal cancer patients undergoing surgery; observational	Overall AL incidence: 14.65% (no intervention vs control groups defined)	Not reported	Multivariate analysis: low postoperative albumin (p = 0.044); high PG-SGA score (p < 0.001); diabetes (p = 0.003); perioperative blood transfusion (p < 0.001); tumour close to the anus (p = 0.004); diarrhoea (p = 0.005)	[29]
Sørensen et al. (1999)	Lifestyle risk factors: smoking status and alcohol abuse assessed preoperatively	Overall AL rate: 15.9% (53/333); higher incidence in smokers and alcohol abusers vs non-smokers/abstainers	Smoking: RR 3.18 (95% CI 1.44-7.00); alcohol abuse: RR 7.18 (95% CI 1.20-43.01)	Statistically significant (multivariable logistic regression)	[32]
Frasson et al. (2016)	Preoperative nutritional status (serum protein/albumin) and surgical technique assessed prospectively; multicentre observational study	Overall AL incidence: 8.4%(93/1102); clinically relevant AL: 6.5%	Preoperative serum protein: OR 0.6 per g/dL (protective); stapled technique: OR 2.1 (clinically relevant AL)	p < 0.0001 (serum protein, overall AL); p = 0.004 (serum protein, clinically relevant AL); p = 0.03 (stapled technique)	[9]
Finco C (2007)	Perioperative enteral immunonutrition enriched with arginine, omega-3 fatty acids, and RNA for 6 days pre-op and resumed post-op day 2 vs low-fibre diet	Not reported	Not reported	Not reported	[23]
Peters et al. (2018)	Perioperative lipid-enriched enteral nutrition via continuous tube feeding from 3-h pre-op to 6-h post-op vs no perioperative nutrition (standard care)	9% (12/132) vs 8% (11/133)	RR 1.01 (95% CI 0.94-1.09)	p = 0.81	[25]
Kwag et al.(2014)	No preoperative nutrition was given; patients only underwent NRS 2002 at admission	9.1% (9/99) nutritional risk vs 3.2% (8/253) no nutritional risk	OR = 3.08, 95% CI 1.15-8.19	p = 0.027	[11]
Brisinda et al. (2022)	No intervention was given, only preoperative assessment of serum albumin, haemoglobin, weight loss, and PNI	18.2% (PNI < 40) vs 6.6% (PNI ≥ 40)	Not reported	PNI: p = 0.0001; serum albumin: p = 0.006; haemoglobin: p = 0.02; preoperative weight loss: p = 0.01	[13]
Do Woong Choi et al. (2023)	Preoperatively, patients were given immunonutrition “Impact” 3 times a day for 1 week. Immunonutrition contained arginine, omega-3 fatty acids, and nucleotides	Overall AL: 4.4% (16/361)	Not reported	Albumin: p = 0.4457; protein: p = 0.6245	[16]
Yamamoto et al. (2020)	Patients were given preoperative enteral nutrition with an elemental diet (no solid food, only water or tea allowed in addition) of 1800-2400 kcal/day for 2-4 weeks. It was given either orally or through a nasogastric tube	4% (1/24) vs 12.5% (3/24)	Not reported	p = 0.04	[30]
Younan et al. (2025)	Preoperative oral immunonutrition supplementation. Two Impact Advanced Recovery (containing arginine, omega 3 fatty acids, and nucleotides) shakes per day, each being 250 mL for 7 days preoperatively and 3 days post-discharge	4.8% (received immunonutrition) vs 5.2% (did not receive immunonutrition)	OR 0.58 (95% CI 0.22-1.56)	p = 0.58	[26]
Guo et al. (2016)	Preoperative nutritional therapy (NT) with enteral nutrition, and an additional parenteral nutrition of calories are not met with enteral alone for 7 days preoperatively	2.3% (1/44) vs 17.9% (7/39)	Not reported	p = 0.023	[27]
Asteria et al. (2008)	No nutritional intervention, preoperative risk factor assessment	Overall AL: 15.2% (79/520)	J-pouch: OR 0.283 (95% CI: 0.086-0.928; p= 0.027); malnutrition: OR 2.803 (95% CI: 1.516-5.182; p < 0.001); preoperative albumin < 3.5 g/dL: OR 2.8 (95% CI: 1.3-5.1; p = 0.03)	AL incidence (p < 0.05) correlated with higher age, obesity, malnutrition, intraoperative contamination, patients with colonic J-pouch reservoir, alcohol/smoking, and lower centre case volume (<20 per year)	[31]
Thornblade et al. (2017)	Oral immunonutrition (Impact) 237 mL, taken 3 times daily for 5 days preoperatively	18.7% (642/3375) vs 81.3% (2743/3375)	SAEs: RR 0.76 (95% CI: 0.49-1.16); prolonged length of stay (PLOS): RR 0.77 (95% CI: 0.58-1.01)	SAE: p = 0.19; PLOS: p = 0.05	[19]
Wei et al. (2024)	No intervention given, assessment of patient risk factors and nutritional status (PNI). Inflammatory markers assessed: NLR, PLR, lymphocyte-to-monocyte ratio (LMR), Systemic Immune-Inflammation Index (SII), pan-immune-inflammation value (PIV), and Systemic Inflammation Response Index (SIRI). Biochemical tumour markers: carcinoembryonic antigen (CEA), CA125 and CA199,	Overall AL: 19.4% (24/124)	Not reported	PNI: p = 0.048	[12]
Shimura et al. (2018)	No nutritional intervention given, patient risk factors assessed, including preoperative serum albumin	Overall AL rate: 5.6% (11/196)	Preoperative serum albumin < 3.2 g/dL: OR 7.53 (95% CI 1.60-55.80)	p = 0.0095	[8]
Telem et al. (2010)	No nutritional intervention given, only measured patient risk factors and preoperative serum albumin	Overall AL: 2.6% (90/3501)	Preoperative albumin < 3.5 g/dL: OR 2.8 (95% CI 1.3-5.1)	Preoperative albumin < 3.5 g/dL: p = 0.03	[4]
Reisinger et al. (2015)	No nutritional intervention given, patients were screened with the Short Nutritional Assessment Questionnaire (SNAQ). Patients who scored 3 or higher were labelled malnourished and required to see a dietician. No duration mentioned	Overall AL: 14.9% (37/249)	High SNAQ score: OR 2.33 (95% CI 0.90-6.00)	SNAQ score: p = 0.08	[17]
Oguz et al. (2007)	Parenteral L-alanine-L-glutamine (1 g/kg/day) was added to enteral nutrition. Both were given before and after surgery for at least 5 days. Patient risk factors were measured and serum albumin for NS	Minor leaks: 2% (1/57) vs 8% (4/52)	Not reported	p > 0.05	[24]
Suding et al. (2008)	No nutritional intervention given, patient risk factors measured and preoperative serum albumin for NS	Overall AL: 3.6% (24/672); low albumin (<3.5 g/dL): 10/143 patients (7%); normal/higher albumin (≥ 3.5 g/dL): 13/519 (2.5%)	Preoperative serum albumin < 3.5 g/dL: OR 2.56 (95% CI 1.07-6.16)	Preoperative albumin < 3.5 g/dL: p = 0.04	[6]

Discussion

AL remains a devastating complication of colorectal surgery, contributing to substantial postoperative morbidity, prolonged hospitalization, reoperation, and mortality. Across the literature, poor nutritional status, particularly preoperative hypoalbuminaemia, emerges as one of the most consistent predictors of AL. A large observational study, with an AL incidence of 5.55%, demonstrated that moderate hypoalbuminaemia was associated with more than a sixfold increase in leak odds, while severe hypoalbuminaemia conferred an almost 25-fold increase [3]. Similarly, in another cohort with a lower baseline AL rate (2.6%), hypoalbuminaemia remained an independent predictor of AL, with patients experiencing a 2.8-fold increase in odds [4]. Similar findings identifying hypoalbuminaemia as an independent risk factor have been reported across diverse colorectal populations [5-8], supporting its value as a clinically accessible marker of impaired physiological reserve rather than an isolated laboratory abnormality.

Beyond serum albumin alone, one study demonstrated that low preoperative serum total protein was the only independent risk factor for overall AL, with each 1 g/dL increase in total protein associated with a 40% reduction in leak odds [9]. Composite nutritional indices further strengthen the association between malnutrition and AL. Lower scores on the Onodera Prognostic Nutritional Index (OPNI), Prognostic Nutritional Index (PNI), and elevated Nutritional Risk Score 2002 (NRS 2002) were associated with significantly higher leak rates and worse postoperative outcomes [10-13]. In a retrospective cohort study, higher OPNI scores were independently associated with a reduced risk of AL, corresponding to an approximately 30% lower odds of leakage per incremental increase in OPNI [10]. On the other hand, a lower PNI was significantly associated with leak development, with an odds ratio of 0.705 (95% CI: 0.641-0.775; p = 0.012) [10].

Two large cohort studies using NRS 2002 tools demonstrated that preoperative nutritional risk is independently associated with AL. Ostermann et al. reported that patients with higher NRS scores had more than double the odds of AL after rectal cancer surgery [14]. Similarly, in a prospective colorectal cancer cohort, patients with an NRS 2002 ≥ 3 experienced significantly higher rates of overall postoperative complications, including AL, with a threefold increase in odds compared with patients not at nutritional risk [11]. Composite inflammatory-nutritional markers, such as the C-reactive protein-to-albumin ratio (CAR), may improve the prediction of postoperative complications. Specifically, each unit increase in CAR was associated with a 4.5% increase in the odds of AL, as well as longer hospital stay and higher postoperative mortality [15]. These findings suggest that combined assessments of nutritional and inflammatory status may outperform single biomarkers in identifying high-risk patients.

However, not all studies were concordant, highlighting important limitations in nutritional risk assessment. Several analyses reported that albumin alone had limited predictive value when evaluated in isolation, particularly in populations with competing risk factors [16]. Broader constructs, such as frailty and sarcopenia, were more strongly associated with postoperative sepsis and overall morbidity rather than AL specifically, suggesting that traditional nutritional biomarkers may be more directly relevant to anastomotic integrity than global geriatric assessments [17].

Preoperative immunonutrition and enteral supplementation were consistently associated with improvements in secondary outcomes, including reduced SSIs, shorter hospital stays, and improved immune or inflammatory profiles [18-21]. Supporting this, one study demonstrated that preoperative enteral immunonutrition effectively prevented SSIs in patients with colorectal cancer who were not malnourished: none of the patients receiving immunonutrition developed an SSI, compared with an overall SSI rate of 14.7% in the control group [22]. In addition, another study reported a positive effect of immunonutrition on immune function, with increased CD4 lymphocyte counts observed both at the time of surgery and throughout the postoperative period in patients receiving immunonutrition [23]. However, reductions in AL incidence were less consistent and often reported as non-significant trends [19,21,24,25], particularly within enhanced recovery after surgery (ERAS)-based pathways where baseline complication rates were low [25,26]. Notably, preoperative nutritional therapy, particularly exclusive enteral nutrition, has been shown to significantly reduce AL rates and the need for temporary stomas in patients with Crohn’s disease, likely through improvements in albumin levels and reductions in systemic inflammation [27]. In contrast, routine preoperative TPN in patients with ulcerative colitis undergoing elective surgery is not supported. The only statistically significant association observed was an increased risk of line-related complications, whereas trends toward higher reoperation rates and ALs in the TPN group did not reach statistical significance [28].

While nutritional status is undeniably a critical and modifiable risk factor for AL, it is important to recognise that AL is a multifactorial complication influenced by a range of perioperative and intraoperative variables. Operative duration ≥ 200 minutes, intraoperative blood loss ≥ 200 mL, perioperative transfusions, and steroid use significantly increased AL risk [4,5,29,30]. Smoking and alcohol excess were also repeatedly identified as independent contributors to leak risk or severity [5,31,32]. Restrictive intraoperative fluid administration has been suggested to reduce postoperative complication rates, including ALs, within ERAS protocols. Patients receiving lower fluid volumes (<0.15 mL/kg/min) had lower overall complication (19% vs 25%) and leak rates (3.2% vs 6.2%); however, these differences did not reach statistical significance, indicating a possible trend toward benefit [33]. More broadly, implementation of ERAS pathways has been associated with substantial reductions in AL, with one cohort demonstrating a decrease from 6% to 1.8% in patients with colorectal cancer and metabolic syndrome, alongside shorter hospital stays, reduced opioid use, earlier return of bowel function, and fewer reoperations [34].

The impact of anastomotic technique, particularly stapled versus hand-sewn anastomosis, on AL risk remains heterogeneous across studies. An analysis reported no significant difference in leak rates between stapled and hand-sewn techniques or between anastomotic configurations, suggesting that technique alone may not be a dominant determinant of AL [5]. In contrast, other cohort studies identified stapled anastomoses as an independent risk factor for clinically relevant leaks, even after adjustment for confounders [9,32]. These findings challenge earlier meta-analyses and suggest that stapling may confer increased risk in specific clinical contexts or patient populations. Notably, adjunctive technical strategies, such as seromuscular reinforcement of the anastomotic line, were associated with significantly reduced leak rates and increased adoption over time, which coincided with improved outcomes, indicating that technical refinement rather than technique selection alone may be more important in mitigating leak risk [5].

## Conclusions

This systematic review supports a substantial body of evidence demonstrating that preoperative nutritional status plays a critical role in predicting AL following colorectal surgery. Across multiple studies, hypoalbuminaemia and markers of malnutrition were consistently associated with increased leak risk, although not all analyses found albumin alone to be a reliable predictor, reflecting ongoing debate regarding the most accurate and clinically useful nutritional markers. These findings highlight the importance of interpreting albumin as a surrogate of physiological reserve rather than an isolated biochemical value. AL is multifactorial, with perioperative factors such as prolonged operative time, blood loss, transfusion, and steroid use further contributing to risk. The interaction between nutritional deficits and operative stressors likely amplifies vulnerability, particularly in complex or high-risk patients. 

Interpretation of current evidence is limited by heterogeneity in leak definitions, diagnostic methods, and study design. Targeted nutritional interventions, including immunonutrition and perioperative protein supplementation, have consistently improved secondary outcomes such as infection rates and hospital stay, though their effect on leak prevention remains less clear. Reducing leak incidence will require a multidisciplinary approach, combining early nutritional optimisation with careful surgical technique and comprehensive perioperative management. Integrating nutritional assessment into standard perioperative pathways may represent a practical, modifiable strategy to improve outcomes in colorectal surgery. 
